# Role of nutritional and metabolic status on the pullet to hen transition and lifetime productivity

**DOI:** 10.3389/fphys.2025.1585645

**Published:** 2025-06-06

**Authors:** Thiago L. Noetzold, Martin J. Zuidhof

**Affiliations:** Department of Agricultural, Food and Nutritional Science, University of Alberta, Edmonton, AB, Canada

**Keywords:** laying hens, broiler breeders, carcass composition, fatness, feed intake

## Abstract

Modern poultry industry is divided into two main commercial categories: laying hens (egg-type) and broiler breeders (meat-type). They have been selected differently over the past 100 years. Despite the difference in egg production between egg- and meat-type chickens, similar physiological triggers are attributed to both during the transition from pullet to hen. In addition to the photoperiod threshold, reproduction in chickens is also connected to three main metabolic thresholds: nutrient intake, body weight (**BW**), and carcass composition. Much is still unknown regarding the physiological effects of these thresholds. Many differences in management and nutritional strategies have been attributed to egg- and meat-type chickens. However, not a lot of emphasis has been put on the possible physiological similarities between the main metabolic factors (body composition and nutrient intake) and thresholds (critical BW and body fat) affecting egg- and meat-type pullets during the transition to the reproduction period. Therefore, this review summarized current knowledge on the metabolic status of the pullets affecting the onset of sexual maturation, focusing on its integration with photoperiodic cues and reproductive physiology, and how the latter is affected by the metabolic status of egg- and meat-type chickens.

## 1 Introduction

The pullet to hen transition period is a critical stage of development that can determine chicken reproductive success. During this period, chickens initiate sexual maturation, transitioning from immature and growing phase to a mature and reproducing phase, where several factors are important. The first and most widely factor is photoperiod (Whetham, 1933). The proportion of diurnal photo (light) and scoto (dark) period is detected by the avian brain and affects their reproduction (Cassone, 2014). In nature, photoperiod responsiveness of chickens has its basis in seasonality, where increasing photoperiod is linked to initiation of reproduction. Other seasonal factors besides photoperiod are important in chicken reproduction, such as increased food availability in spring and summer seasons. In this context, metabolic thresholds are important factors in addition to photoperiod that influence the pullet to hen transition. Over the years, there have been three main metabolic thresholds associated with reproduction, and are affected by the nutritional status of chickens: nutrient intake (Hocking, 2004; Noetzold et al., 2025), BW (Plessis Du and Erasmus, 1972; van der Klein et al., 2018a), and carcass composition (Kwakkel et al., 1995; Heijmans et al., 2023; Noetzold et al., 2024; Noetzold et al., 2025). While these metabolic thresholds influence reproduction, the chicken strain utilized is another important factor. The poultry industry is divided into two main commercial categories: egg- and meat-type chickens. They have been selected differently over the past 100 years to have distinct commercial purposes (Punnett and Bailey, 1914; Schnetzler, 1936). Breeding programs for laying hens (egg-type) have been focused on improving egg production by getting birds into longer laying cycles, maximizing profit per hen housed with production cycles that can go up to 100 weeks of age (Bain et al., 2016). On the other hand, broiler breeders (meat-type) have been intensively selected for muscle growth and feed efficiency (Zuidhof et al., 2014). Therefore, greater BW, lower egg production peak, and persistency are characteristics of meat-type birds when compared to laying hens (Caldas et al., 2018). Ultimately, there exists considerable egg production disparity between egg- and meat-type chickens, approximately 190 vs. 500 eggs per cycle in laying hens and broiler breeders, respectively (Lohmann, 2018; Hy-line, 2019; Cobb-Vantress, 2020; Aviagen, 2021).

**FIGURE 1:**
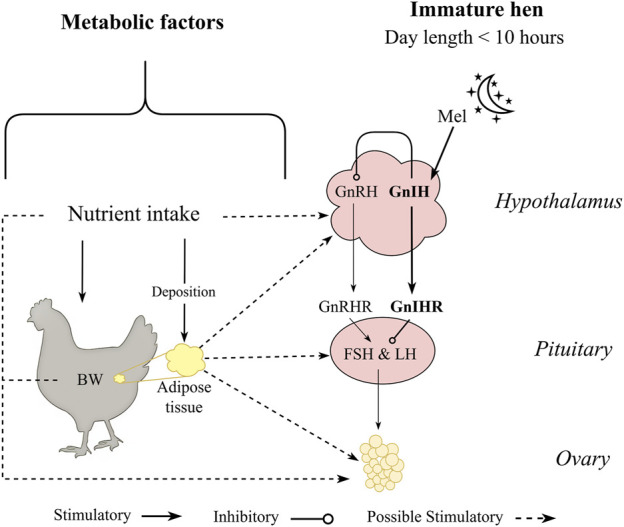
Metabolic factors hypothesized to influence sexual maturation of pullets maintained in a non-stimulatory photoperiod. Pullets maintained at photoperiod equal or lower than 10 h have gonadotropin inhibitory hormone (GnIH) as the primary neuropeptide release by the hypothalamus in response to melatonin (Mel) produced by the pineal gland during the scotophase. Birds are maintained under an immature state by GnIH and its receptor (GnIHR) at the anterior pituitary gland, suppressing hormonal cascade of follicle stimulating hormone (FSH) and luteinizing hormone (LH). However, the transition period from pullet to hen can occur without any photostimulation (photoperiod equal or greater than 12 h) and are hypothesized to be influenced by nutritional status and carcass adipose composition.

Despite the difference in egg production between laying hens and broiler breeders, similar physiological triggers are attributed to both categories, especially related to the onset of lay. As mentioned, photoperiod influences reproductive output, with early studies showing an increase in day length coinciding with the initiation of reproduction in birds (Whetham, 1933; Byerly and Moore, 1941) and that was well characterized in broiler breeders and laying hens over the past years (Lewis et al., 2004; Sharp, 2005; Lewis, 2006; Hanlon et al., 2020). In addition to the photoperiod signal, laying hens and broiler breeders start sexual maturation through a metabolic signal (van der Klein et al., 2018a; Zuidhof, 2018; Baxter and Bédécarrats, 2019). Both broiler breeders and laying hens had the transition period occurring before the photoperiod signal when birds were fed *ad libitum* during the rearing period (Baxter and Bédécarrats, 2019; Zukiwsky et al., 2021; Carney et al., 2022). Additionally, after genetic selection in laying hens, photorefractoriness was practically eliminated from egg-type chickens (Morris and Sharp, 1995), which has brought into focus other factors affecting reproductive physiology in laying hens, such as nutrient intake and body composition (Figure 1). Taking this together, a link between reproductive physiology and metabolic thresholds (nutrient intake, BW, and carcass fat composition) during the pullet to hen transition period in egg- and meat-type chickens is suggested even when birds are maintained in a non-stimulatory photoperiod (Figure 1).

To the authors knowledge, not a lot of emphasis has been put on the possible physiological similarities between the main metabolic factors (body composition and nutrient intake) and thresholds (BW and body fat) affecting egg- and meat-type pullets and their transition to the reproduction period. This review aimed to summarize current knowledge on the metabolic status of the pullets affecting the onset of sexual maturation, focusing on its integration with photoperiodic cues and reproductive physiology, and how the latter might be affected by the metabolic status of egg- and meat-type chickens.

## 2 Genetic selection of meat and egg-type chickens

**FIGURE 2:**
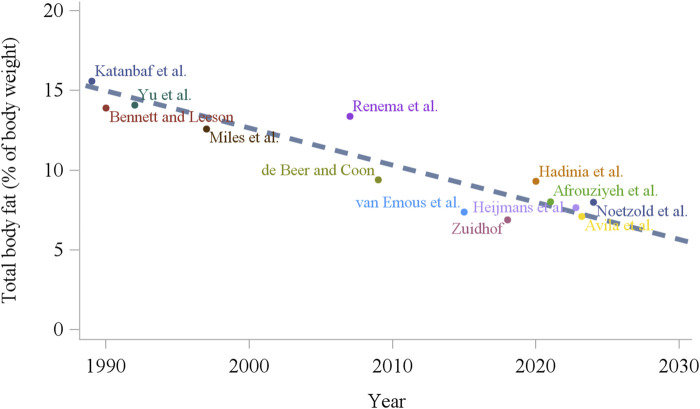
Total body fat content of broiler breeders around the time of photostimulation age (20–22 weeks) over the last 30 years (Katanbaf et al., 1989; Bennett and Leeson, 1990; Yu et al., 1992; Miles et al., 1997; Renema et al., 2007; de Beer and Coon, 2009; van Emous et al., 2015; Zuidhof, 2018; Hadinia et al., 2020; Afrouziyeh et al., 2021b; Avila et al., 2023; Heijmans et al., 2023; Noetzold et al., 2024).

**FIGURE 3:**
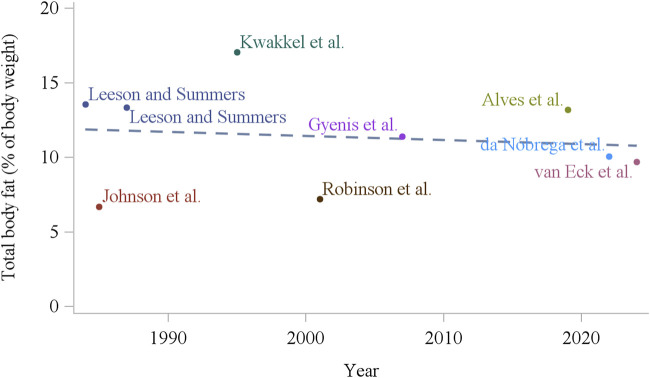
Total body fat content of white laying hens (16–18 weeks of age) over the last 40 years (Lesson and Summers, 1984; Johnson et al., 1985; Summers et al., 1987; Kwakkel et al., 1995; Robinson et al., 2001; Gyenis et al., 2007; Alves et al., 2019; da Nóbrega et al., 2022; van Eck et al., 2024).

In the primary broiler breeder companies, genetic selection of the male line has been made primarily by improving growth and efficiency (Hocking and McCorquodale, 2008), and in the female line by improving reproductive and growth traits (Pollock, 1999), which are two negatively correlated factors (Siegel and Dunnington, 1985; Robinson et al., 1993a). Zuidhof et al. (2014) in a study using commercial broiler strains unselected since 1957, 1978, and 2005, have shown that from 1957 to 2005, growth rate of 56 day old progeny increased by over 400%. The authors linked the results to carcass conformation where breast yield has significantly increased. Also, the efficiency of these birds increased considerably, with a 50% reduction in feed conversion ratio from 1957 to 2005 at 42 days (Zuidhof et al., 2014). Importantly, broiler breeders carry this genetic potential, and as shown by Carney et al. (2022), over the last 60 years, genetic selection of meat-type parent stock has increased the yield of specific meat portions, growth potential, and efficiency. Together, an increased degree of feed restriction and genetic selection for growth and efficiency (Zuidhof et al., 2014), modern broiler breeders have become leaner over the years, with a decrease of approximately 50% in body fat over the last 30 years (Figure 2; Eitan et al., 2014; Zuidhof, 2018). This may have unintended consequences on how we feed and manage modern broiler breeders. Laying hens, on the other hand, have been selected mainly for reproductive traits (Bain et al., 2016), making laying hens perform considerably well in egg production. Although low fat deposition is not a problem in modern laying hens, its role is not well understood. Additionally, there was no clear change in total fat deposition over the last 40 years (Figure 3), which suggests that fat deposition was not affected by genetic selection for reproductive efficiency in egg-type chickens.

Feed intake capacity and voracity are two of the main differences between egg- and meat-type chickens. Selection traits of BW and meat yield in broilers and increased requirements for growth and development have been reported as the reason for their increased feed intake (Emmans, 1987; Richards et al., 2010). On the other hand, feed intake itself is considered the stimulant for high growth rates (Marks, 1979; Emmans, 1987). Therefore, because of nutrient demands and feed intake capacity, greater feed intake occurs in broiler breeders. When allowed full access to feed, broiler breeders become rapidly overweight (Heck et al., 2004), whereas overfeeding in laying hens is generally not a practical problem (Richards et al., 2010). This means that at 42 days of age, unrestricted broiler breeder hens weigh around 5-times the laying hens (Zhao et al., 2004). Such differences in BW can lead to several differences in physiological responses. Compared to layers, broiler breeders have reduced cardiopulmonary capacity, because of their higher muscle mass relative to body weight (Hafez and Hauck, 2005). To compensate for this high energy demand, the circulatory system must perform at a higher rate in order to supply sufficient oxygen to the muscle tissues when compared to layers (Wideman et al., 2010; Ho et al., 2011). This predisposes meat-type chickens to metabolic disorders, such as heart failure and ascites (Wideman et al., 2010). On the laying industry side, intense selection for earlier age at sexual maturation, higher peak production, and longer laying persistency have different consequences. The high egg production rates can lead to metabolic disorders such as fatty liver hemorrhagic syndrome (**FLHS**; Wolford and Polin, 1972) and osteoporosis (Sandilands, 2011) due to the large amount of lipid metabolized in the liver for yolk formation and high calcium requirement for eggshell formation, respectively. Studies on dietary calcium, energy to protein ratio, and pullet rearing strategies for laying hens have been developed seeking to decrease these metabolic problems (Yousefi et al., 2005; Rozenboim et al., 2016; Hervo et al., 2021). Regardless, it is still reported that laying hens suffer from bone fractures up to 30% during the production cycle (Johnsson et al., 2015) which can increase as they approach the end of the laying cycle (Alfonso-Carrillo et al., 2021). Additionally, fatty liver hemorrhagic syndrome is still one of the main mortality causes in commercial laying hens kept in cage systems (Shini et al., 2019). In the following sections, we contrast the main differences and similarities between egg- and meat-type chickens related to nutritional and metabolic influences in their reproductive outcome.

### 2.1 Follicular development

An important genetic consideration for the production potential of commercial lines is ovarian follicle development. In commercial avian species, only the left ovary and oviduct develop at puberty whereas the right remains rudimentary (Hrabia et al., 2022). In an ideal scenario, commercial mature hens would produce one good quality egg per day. In this scenario, egg-type chickens are more efficient than meat-type birds, with approximately 320 eggs laid in 365 days of production (Lohmann, 2018; Hy-Line, 2019), compared to around 180 eggs in meat-type chickens in the same period (Cobb-Vantress, 2020; Aviagen, 2021). This difference is associated with several factors, such as earlier onset of lay, higher peak production, and absence of photorefractoriness in egg-type chickens. Additionally, *ad libitum* fed laying hens can still have a well-defined and organized follicular hierarchy, with a uniform follicular selection to the rapid growth phase (one per day) when compared to the broiler breeder hens (Johnson, 2012). Nonetheless, genetic selection might have improved follicular hierarchy stability in meat-type chickens over the last decades (Noetzold et al., 2024).

Domestic hens lay eggs in sequences (consecutive days of oviposition) and pause days separate the sequences. The longer the sequences, the lower the number of pause days, and consequent greater egg production. Therefore, consistent follicle recruitment to the rapid growth phase and the time that the follicles take to mature might be key factors that differentiate reproductive performance between laying hens and broiler breeders. To reach the ovulation point, the follicle undergoes a long process that starts when the hen is still in the embryonic phase (incubation period). The ovary of commercial chickens presents approximately 12,000 follicles at the time of hatch (Johnson, 2014) and less than 600 will further develop to the next follicular phases through follicular recruitment and maturation. There are two main follicular recruitment processes that are important for the chicken oocyte to reach maturity: primordial follicular recruitment and pre-hierarchical recruitment to the rapid growth phase (pre-ovulatory phase). The former starts when primordial follicles in the ovary obtain the ability to respond to follicle-stimulating hormone (**FSH**; Stephens and Johnson, 2020). Two main cellular layers surround the follicle: granulosa cells, already present in the primordial follicles; and theca cells, further divided into internal and external layers (Johnson and Scanes, 2015a; Johnson, 2015b). The granulosa cells of developing follicles begin to express FSH receptors (**FSHR**). It is unknown what mechanism makes the primordial follicles responsive to FSH, since pre-hierarchical follicles do not respond to FSH until later stages of development (Johnson and Lee, 2016). Essentially, activated primordial follicles from the ovary grow to their first stage and develop the theca internal layer, associated with uptake of small amounts of white yolk (Johnson and Scanes, 2015a). The next follicular phase (primary follicles) can remain quiescent for months or years (Ting and Zelinski, 2017). Many hypotheses have been proposed to explain primordial follicular development, but its mechanism has not been fully elucidated. Growth factors might stimulate or inhibit the number of small follicles growing in the ovary of the hen. In mammals, early follicular development is independent from the hypothalamus-pituitary-gonadal (**HPG**) axis, largely regulated by other mechanisms (Pangas and Rajkovic, 2006). Insulin like growth factor I and II (**IGF-I; IGF-II**), Anti-Müllerian Hormone (**AMH**), and the 5′-Adenosine Monophosphate-Activated Protein Kinase pathway (**AMPK**) have been related to regulate primordial follicular activation (Fortune, 2003; Zhao et al., 2021). For instance, a combination of decreasing inhibitory influences (e.g., AMH and melatonin) have been reported as possible factors for early follicular development in mice (Durlinger et al., 2001; 2002; Jang et al., 2016). In birds, when leptin was administered intraperitoneally in 7 day old female layers, ovary gene expressions of IGF-I and IGF-I receptor were increased, as well as a decrease of ovary AMH expression (Ahmadi and Ohkubo, 2022). Additionally, AMH receptor decreased expression as the follicles increased in size (follicles from 1 to 3 mm), mostly expressed in granulosa cells of the smallest follicles (Lemcke et al., 2018). Melatonin (**MEL**) was also reported as a potential inhibitor of gonadotropin receptors at the level of primordial follicles (Liu et al., 2017). However, the first follicle activation mechanism is still unclear in chickens and needs further research. In the subsequent phases, lipoprotein accumulates in the primary follicles and pre-hierarchical follicles begin to appear. The pre-hierarchical follicles can be further divided by size: 3–5 mm, large white follicles (**LWF**); and 6–8 mm, small yellow follicles (**SYF**; Johnson, 2012).

The second important follicular recruitment occurs when follicles are selected from a pool of SYF (the largest pre-hierarchical follicles) to a rapid growth phase–pre-ovulatory follicles or large yellow follicles (**LYF**). This is a rate limiting process and is crucial for the development of mature oocytes. In this phase, the follicle accumulates large amounts of yolk, composed mainly of yolk-targeted very low lipoprotein (**VLDLy**) and vitellogenin (**VTG**; Walzem, 1996). To maximize egg production by having one egg per day, the time between oviposition should be close to or less than 24 h, avoiding pause days. The pre-ovulatory follicles in the ovary (>10 mm) are also classified by their size, where the largest follicle (**F1**) is the next one into the ovulation sequence followed by the second largest (**F2**) and so forth. Ideally, 6 to 8 follicles in the rapid growth phase would result in one egg per day being laid. Hence, the maturation rate of these follicles is also important. As long as one follicle is recruited to the rapid growth phase daily, and follicles take around 7 days to mature, one egg would be laid per day.

Once in the rapid growth phase, follicles from a modern egg-type chicken line took approximately 7.4 days to mature and ovulate (McLeod et al., 2014) when compared to 8.0 days in unselected lines (McLeod et al., 2014) with similar duration from hens in the early 1980s (8.0 days; Zakaria et al., 1983). In the meat-type chickens, the pre-ovulatory follicles might spend more time in the rapid growth phase (maturation) when compared to egg-type chickens. Studies from the 1990s and 2000s showed that follicles in the rapid growth phase from broiler breeders have taken approximately 8.4 days and 8.8 days to ovulate, respectively (Yu et al., 1992; Bruggeman et al., 2005). No updated follicular maturation rate data is published for modern broiler breeder hens. In laying hens, genetic selection for egg number is generally positively correlated with longer sequences and a lower number of sequences (Wolc et al., 2019). Faster yolk deposition in the pre-ovulatory phase from modern laying hens is also correlated with their genetic advancement (Song et al., 2023). Therefore, this implies a highly efficient and organized follicular development in egg-type chickens and might be one of the key genetic differences when compared to meat-type chickens. Apart from the genetic differences, additional discussion regarding an interaction between feeding strategies, BW, and carcass composition on reproductive physiology are presented in subsequent sections.

## 3 Photoperiodic control

### 3.1 Photoperiod regulation and reproductive axis physiology

Photoperiod dynamics in commercial chickens have been extensively shown elsewhere (van der Klein et al., 2020; Hanlon et al., 2020) and are succinctly described below. Chicken reproductive status is responsive to positive photoperiods, which means they will respond to increasing photoperiods to activate their reproductive axis. Commercial poultry is photostimulated (increase light intensity and duration) at a specific age and it varies according to their genetic breed. Additionally, selection of photostimulation age is linked to productivity (egg production). It occurs around 16–18 weeks of age for laying hens (Lohmann, 2018; Hy-line, 2019), and between 20 and 23 weeks for broiler breeders (Cobb-Vantress, 2020; Aviagen, 2023). This is related to the hypothalamic age maturity, when birds are responsive to the increasing photoperiod stimulus. Afterwards, they will commence egg production (Shi et al., 2020). The light stimulus is detected by photoreceptors (Dunn and Sharp, 1990), presented in the eye, pineal gland, and hypothalamus (Kumar et al., 2004), decreasing the levels of MEL and translating the photoperiodic signal into a neuroendocrine signal (Saldanha et al., 2001). Both responses together initiate sexual maturation, where the hypothalamus coordinates the initiation of the HGP axis by producing and releasing gonadotropin releasing hormone (**GnRH**; Bédécarrats, 2015). During non-stimulatory photoperiod, gonadotropin inhibitory hormone (**GnIH**) produced in the hypothalamus maintains the birds sexually immature in response to MEL produced through the pineal gland (Ubuka et al., 2005), by stimulating the gonadotropin-inhibitory hormone receptor (**GnIH-R**) in the pituitary gland, preventing the release of FSH and luteinizing hormone (**LH**; Ubuka et al., 2006; Maddineni et al., 2008). Once birds are photostimulated, GnRH stimulates its receptor in the pituitary gland, which in turn stimulates the production and release of LH and FSH (Bédécarrats et al., 2016). The gonadotrophins (LH and FSH) act at the ovary level to produce sex hormones and initiate sexual maturation (Robinson et al., 2003; Bédécarrats, 2015). Once follicles are responsive to the gonadotrophins, estradiol (**E**_**2**_) is produced by the theca layer of small white follicles (**SWF**) in response to increased FSH (Robinson and Etches, 1986; Johnson and Woods, 2009) and progesterone (**P**_**4**_) is produced by the granulosa cells in response to increased LH (Johnson, 1990). While the major role of LH is ovulation through the positive feedback between P4 and GnRH, FSH is responsible mainly for the follicular maturation process. Estradiol exerts negative feedback on gonadotrophins in chickens (Hanlon et al., 2022) while P_4_ has positive feedback and triggers LH surge towards ovulation (Robinson et al., 1998).

### 3.2 Hypothalamo-pituitary-gonodal axis maturation (age)

Sexual maturation is important for egg production in chickens. Earlier or late onset of lay changes egg production output because it can increase or decrease the number of eggs at the end of the production cycle. Egg size and egg laying persistency are always considered when it comes to earlier or late onset of lay. In this regard, the onset of lay is commonly determined by photostimulation, but also might be influenced by metabolic factors. Hypothalamus responsiveness to photostimulation is age specific, which means that before a certain age (critical age threshold) where the HPG axis cannot be activated by the light stimulus (Shi et al., 2020). It has been reported that 14–16 weeks of age is the critical age for broiler breeders (Bruggeman et al., 1998; Ciacciariello and Gous, 2005). In laying hens, surges of LH were reported in birds photostimulated at 6 weeks of age, which indicates hypothalamus responsiveness (Lewis et al., 1994). Nonetheless, the average photostimulation age recommended for commercial laying hens and broiler breeders is generally later (between 16 to 18 and 20–23 weeks of age, respectively) because production performance is maximized. As noted, common age for photostimulation in both egg- and meat-type chickens is later than the critical age related to the hypothalamic maturation (photoperiod responsiveness age). This is because responsiveness to photostimulation is not the only physiological threshold to reproduction. Body development (BW, body condition, and bone mineralization), for example, is not completed when the hypothalamus is mature, and thus, photostimulation is delayed. As discussed in the next sections, the nutritional and metabolic status of birds also interferes with the reproductive axis, and therefore, with the reproductive output of commercial poultry.

## 4 Integration of metabolic control to reproductive physiology

### 4.1 Feed intake

In meat-type chickens, feed restriction programs have been extensively used to control their BW, preventing broiler breeders from getting overweight and having dysfunctional follicular development. Thus, feed restriction is a common feeding strategy that regulates the metabolism of meat-type chickens, and ultimately improves their reproductive output. In the past, several studies have shown impaired reproductive performance when *ad libitum* feeding was used in broiler breeders (Hocking, 1993; Robinson et al., 1993b; Renema and Robinson, 2004; Chen et al., 2006). On the other hand, despite the possible predisposition to FLHS, overfeeding egg-type birds is not much of a problem and feed restriction is not commonly used in commercial settings. Recently, several studies evaluated the effects of restricted feeding in laying hens (Hao et al., 2019; Anene et al., 2023; Bahry et al., 2023; Noetzold et al., 2023; Noetzold et al., 2025) which despite its limited commercial application, can provide important information on the metabolic response of laying hens to feed restriction. Although in the long run the reproductive output of *ad libitum* meat-type chickens is poorer than feed restricted birds, full-fed broiler breeders come into production before the photostimulation signal (Heck et al., 2004; Zukiwsky et al., 2021; Carney et al., 2022) and have their sexual maturity age advanced compared to restricted breeders (Renema et al., 1999). This is also observed in laying hens, where birds fed *ad libitum* come into production before photostimulation compared to feed restricted laying pullets (Baxter and Bédécarrats, 2019; Noetzold et al., 2025). This indicates that the underlying metabolic mechanisms are probably highly conserved and therefore similar in egg- and meat-type chickens but usually masked due to line-specific genetic potential and management.

Feed intake control in chickens has been studied extensively and its detailed physiological pathways are reported elsewhere (Richards, 2003; Richards et al., 2010; Classen, 2017; Cline et al., 2022). Additionally, signaling molecules related to feed intake control are also hypothesized to be related to the reproductive axis through the melanocortin system (Hanlon et al., 2020), which involves neurons present in the hypothalamus: neuropeptide Y (**NPY**) and agouti-related peptide (**AgRP**) as orexigenic (anabolic effect), and proopiomelanocortin (**POMC**) and cocaine and amphetamine regulated transcript (**CART**) as anorexigenic (catabolic effect). The melanocortin neurons have been localized throughout many regions of the hypothalamus (Gerets et al., 2000; Wang et al., 2001) and have been hypothesized to directly affect GnRH and GnIH neurons.

**FIGURE 4:**
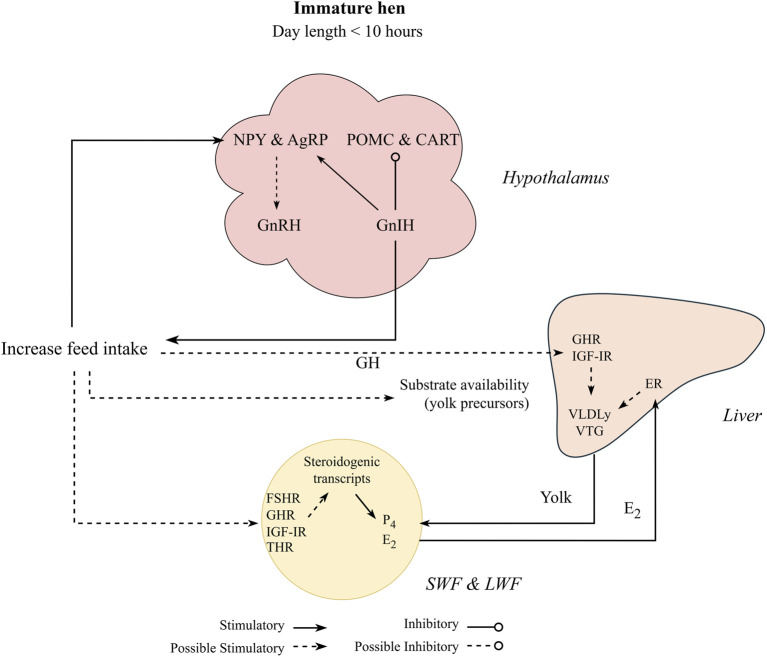
Proposed nutritional effect on pullet to hen transition period, where pullets under non-stimulatory photoperiod initiate sexual maturation through increased feed intake. Gonadotropin inhibitory hormone (GnIH) stimulates the orexigenic peptides (agouti-related peptide, AgRP, and Neuropeptide Y, NPY) and inhibit the anorexigenic peptides (pro-opiomelanocortin, POMC, and cocaine-and amphetamine regulated transcript, CART). Orexigenic factors are reported to stimulate reproduction by stimulating gonadotrophin releasing hormone (GnRH) in response to increased feed intake. Increasing feed intake also stimulates steroidogenesis at the follicular level through activation of insulin-like growth factor I receptor (IGF-IR) and growth hormone receptor (GHR), and potentially follicle stimulating hormone receptor (FSHR) and thyroid hormone receptors (THR). This cascade might initiate follicular growth by stimulating production of estradiol (E_2_) and progesterone (P_4_). E_2_ downregulates the gonadotrophin releasing hormone receptor at the pituitary level and also stimulates yolk precursors (yolk-targeted very low lipoprotein, VLDLy, and vitellogenin, VTG) production through its receptors in the liver (ER-α and β). Additionally, yolk precursors are increased through growth hormone (GH) and its receptor (GHR) activation at the hepatic level.

On the orexigenic side, NPY is speculated to stimulate LH production through GnRH secretion (Figure 4). Hypothalamic injections of NPY in early mature laying hens induced a premature LH surge and increased luteinizing hormone-releasing hormone (LHRH) expression in the median eminence (Contijoch et al., 1993), a region where hypothalamic releasing hormones (neural system) are released to be transported to the anterior pituitary (Dunn et al., 2009). This association between NPY and reproduction was confirmed with earlier sexual maturation in broiler males (6 weeks of age) administered NPY intracerebroventricularly (Fraley and Kuenzel, 1993) and may be linked to greater egg production of native meat-type chickens (Li et al., 2009). When compared to NPY, AgRP expression and its relation to reproduction in chickens has not been widely reported and its role in reproduction remains unclear. Dunn et al. (2015) showed no change in AgRP expression levels after laying hens were released from feed restriction around 25 weeks of age. In mice, increased AgRP related to starvation is responsible for stopping or delaying reproduction (Wu et al., 2012; Padilla et al., 2017). The authors did not indicate direct contact with the GnRH neurons (Padilla et al., 2017), but possibly through leptin receptors presented in the AgRP neurons (see fatness section).

In the anorexigenic pathway, POMC is involved in reproduction, with limited CART literature in chickens. While CART has a suppressive role in reproductive functions in mammals (Lebrethon et al., 2000; Sen et al., 2007), it still needs to be further investigated in chickens. Hypothalamic POMC mRNA levels were increased in the pullet to hen transition period from 22 to 23 weeks of age when compared to more mature broiler breeders (25 and 26 weeks of age; Hadinia et al., 2020). Furthermore, in the same study, POMC expression was greater in breeders with high energy intake than with low energy intake from 22 to 26 weeks of age. Additionally, greater expression of GnRH along the hypothalamus with no effect on GnIH and GnIH-R (pituitary) expression was observed in breeders with high energy intake compared to low energy intake (Hadinia et al., 2020). The authors hypothesized that POMC might modulate GnRH expression, but no direction (upregulation or downregulation) was specified. This result indicates that greater nutrient intake (energy intake) can accelerate puberty in breeder pullets (Figure 4). Other studies related to the melanocortin system have important insights on younger pullets (Dunn et al., 2013; Dixon et al., 2022) and older hens (Ciccone et al., 2007; Xin et al., 2022), but limited information is available on the pullet to hen transition period, indicating a gap in avian literature.

In addition to possible interaction with GnRH, the melanocortin system might be associated with the GnIH neurons. The main hypothesis is that GnIH stimulates feeding behavior in chickens (Tachibana et al., 2005). Intracerebroventricular GnIH injections increased feed intake of layer pullets, associated with increased and decreased expressions of NPY and POMC, respectively (McConn et al., 2014). As mentioned previously, GnIH is known to suppress reproduction during non-stimulatory photoperiods. In addition, more detailed reviews have proposed that GnIH is a key neuron, which regulates reproduction based on the available resources, modulating feed intake and metabolism of mammals and chickens (Tsutsui et al., 2010; Bédécarrats et al., 2022). This modulation might act differently before and after sexual maturation.

As expected, most studies showed that feed restriction increased orexigenic (NPY and AgRP) and decreased anorexigenic (POMC and CART) expressions in chickens and mammals (Song et al., 2012; Sun et al., 2021; Dixon et al., 2022). Interestingly, severe feed restriction is reported to delay or inhibit reproduction in mammals (Sun et al., 2021) and in chickens (Hurwitz and Plavnik, 1989). This is contrary to the results mentioned previously, where greater NPY expression was connected to upregulate reproduction. This example and other inconsistent results on the relationship between melanocortin system and reproduction might have several explanations. First, expression of both orexigenic and anorexigenic pathways is influenced by temporal dynamics, such as age (young vs. older animals), within few days or weeks (Huang et al., 2010), and possibly in a diurnal manner (Stütz et al., 2007). Second, several studies confirmed that *ad libitum* feeding increased GnRH expression (Ciccone et al., 2007; Hadinia et al., 2020), which was one of the factors attributed to advanced sexual maturation or greater egg production in these studies. However, the mechanism behind the GnRH increases is not completely understood. Lastly, correlation between melanocortin system and reproduction cannot be linked to causation. It is possible that in addition to changes in the melanocortin system (hypothalamus), feed intake might also influence reproduction at the ovarian follicular level during the pullet to hen transition. These changes might initiate a hormonal cascade (estradiol feedback to hypothalamus) and influence GnRH levels, or through an unknown route.

The effect of feed intake on follicular recruitment is very complex, and much of the information is still unknown. Studies have been consistent over the years, where follicular recruitment and growth were delayed with feed restriction during the pullet to hen transition period in broiler breeders (Hocking and Robertson, 2000; Diaz and Anthony, 2013; Stephens and Johnson, 2017; Anthony et al., 2022; Stephens et al., 2022) and laying hens (Stephens and Johnson, 2017; Bornelöv et al., 2018; Bahry et al., 2023), and consequently, delayed sexual maturity (Noetzold et al., 2025). Restricting feed intake in modern white and brown laying hens at 80% of the target BW delayed sexual maturation age and restricted hens had fewer SWF compared to *ad libitum* fed birds (Bahry et al., 2023). Similar results were found in LYF number and sexual maturation of brown laying hens by Noetzold et al. (2025). Most of these studies compared at least one *ad libitum* feeding treatment to a feed restricted group, and the degree of feed restriction varied depending on the trial objective. Overall, follicular development differences were more evident when a wider nutrient intake level between groups was utilized. Several metabolic pathways are associated with follicular recruitment, and it has been extensively discussed by Johnson, 2012; Johnson, 2014; Johnson and Scanes, 2015a; Johnson, 2015b). Among the possible mechanisms that feed intake might affect follicular development is through factors linked to the synthesis of steroid hormones (steroidogenesis) from cholesterol (Figure 4). This steroid synthesis occurs at the follicular level (Robinson and Etches, 1986), specifically in the granulosa and theca cells in response to FSH and LH (Ghanem and Johnson, 2019). The abundance of steroidogenic transcripts such as cytochrome P450 family 11 subfamily A member 1 (**Cyp11a1**), cytochrome P450 family 19 subfamily A member 1 (**Cyp19a1**), hydroxy-delta-5-steroid dehydrogenase, 3 beta- and steroid delta-isomerase 1 (**Hsd3b1**), and steroidogenic acute regulatory protein (**STAR**) are increased in follicles of 1 mm in diameter compared to 0.5 mm follicles (Diaz et al., 2011). This suggests that the initial recruitment and growth of primordial and primary follicles depend on steroidogenic transcripts.

Several mechanisms are hypothesized to decrease the abundance of steroidogenic transcripts with feed restriction. One common pathway is through the thyroid hormones. Triiodothyronine (**T**_**3**_) administration has decreased LH and steroids concentration in chicken blood (Sechman, 2013), and feed restriction decreased the levels of circulating T_3_ in immature (Reyns et al., 2002) and mature chickens (Stephens et al., 2022). Despite indication of a negative effect on reproduction, greater plasma T_3_ levels were observed in *ad libitum* fed birds alongside advanced development of LWF, SYF, and LYF, but lower egg production during a period of 6 weeks (Stephens et al., 2022). Sun et al. (2006) observed greater T_3_ levels in *ad libitum* fed pullets compared to restricted fed pullets at photostimulation age, with no changes in T_3_ levels at sexual maturation and at 36 weeks of age. Additionally, lower plasma T_3_ concentration was observed in turkey hens with high egg production than with low egg production, and no changes in the thyroid hormone receptors (**THR**; Brady et al., 2021). The authors indicated that THR in the ovary were not likely to be involved in steroidogenesis in turkeys (Brady et al., 2021). These contradictory results demonstrated that further research is needed to explain not only plasma thyroid hormones concentration, but also its receptors in the HPG axis and liver of chickens. Another mechanism that might decrease the abundance of steroidogenic transcripts through feed restriction is associated with growth factors. Growth hormone receptor (**GHR**) and IGF-I expression in the liver were lower in restricted broiler breeders (for 6 weeks starting at 38 weeks of age) when compared to *ad libitum* feeding (Stephens et al., 2022). While growth hormone (**GH**) stimulates production of yolk proteins in the liver (Hrabia, 2015) and upregulates steroidogenesis in the ovary (Ahumada-Solórzano et al., 2012), IGF-I enhances steroidogenesis directly at the follicular level in chickens (Onagbesan et al., 1999), significantly increasing STAR and Cyp11a1 expressions in the granulosa cells of SYF (Francoeur et al., 2021; 2023). Also, feed restriction can decrease follicular development by reducing yolk substrates produced in the liver. Feed restriction decreased liver lipogenesis, plasma triglycerides and cholesterol (Rajman et al., 2006; Zaefarian et al., 2019) and, hence, delaying sexual development by restricting initial yolk deposition (see fatness section).

In addition to nutrient intake level, feeding frequency and feeding time might affect the metabolic status of chickens. Zuidhof, (2018) fed grandparent breeder hens (male line) using a precision feeding system (birds fed several meals throughout the day), and observed that females with recommended BW had a lower egg production compared to conventionally fed birds on a daily feeding program. In an attempt to increase egg production, BW target was increased after 43 weeks of age, which increased egg production after 45 weeks of age. This increase occurred because several birds were not sexually mature at 45 weeks of age, likely due to insufficient carcass fat reserves. Similar results were found in broiler breeder parent stock, where birds fed multiple times during the day demonstrated lower egg production than pullets 22% heavier at the photostimulation age (van der Klein et al., 2018a). These results were attributed to the feeding pattern from the precision feeding system, where birds fed multiple times during the day deposited less adipose tissue. The hypothesis from the above results indicates that with the increasing genetic potential for growth and lean deposition, meat-type chickens might reach a biological limit regarding fat deposition, where Figure 2 shows total body fat has decreased over the last 30 years. In the above studies, the feeding protocol of multiple meals throughout the day appears to have intensified the lean deposition of breeders at the expense of fat deposition and consequently, impaired their reproductive performance. Further evidence for this hypothesis is shown by Afrouziyeh et al. (2021b) and Noetzold et al. (2024), where relaxing feed restriction of broiler breeders during specific periods has shown to increase egg production and body fat deposition, respectively. Therefore, feed intake appears to influence reproduction more evidently in broiler breeders when compared to laying hens. Nonetheless, feeding level studies have made a significant contribution to understanding the physiology and metabolism of both meat- and egg-type chickens, having a key role in future management decisions.

### 4.2 Body weight and composition

The influence of BW on sexual maturation is not a new concept. In fact, it has been reported long ago (Hays, 1933). This relationship between BW and the pullet to hen transition has been well reported in both egg- and meat-type chickens. When examining BW independently from other factors, a minimum BW for sexual maturation has been established in laying hens and broiler breeders (Lewis et al., 2007). Although the BW can differ between strains, BW of 2.1–2.2 kg in broiler breeder hens (Lewis and Gous, 2006) and 1.4–1.6 kg in laying hens (Dunnington and Siegel, 1984; Lewis et al., 1997) were recommended as minimum for puberty. Laying hens seem to have a consistent BW at sexual maturation in the past decades with few changes within the same breed when fed *ad libitum* (Shi et al., 2020; Hanlon et al., 2021). Interestingly, feed restricted egg-type pullets that achieved lower BW around the transition period had delayed sexual maturation compared to *ad libitum* fed pullets (Summers et al., 1991; Bahry et al., 2023; Noetzold et al., 2025). In meat-type chickens, feed restriction is a common practice, and therefore they have a recommended target BW provided by the primary breeders for optimal reproduction (Cobb-Vantress, 2020; Aviagen, 2023) and these BW targets are generally updated on a 4-year basis. Recent studies have suggested that the recommended target BW around the time of photostimulation (±2 kg at 21 weeks of age) resulted in low performance compared to heavier hens (van der Klein et al., 2018a; 2018b). It is interesting that despite the differences in feeding management practices between laying hens and broiler breeders, decreased BW of egg-pullets and severe feed restriction in meat-type pullets around the transition period negatively impacts reproduction of both categories. Similarly, a common commercial practice of both groups is to delay the moment of photostimulation in flocks that have BW uniformity issues. This is done to allow the smallest pullets in the flock to reach a critical BW and increase reproductive performance.

From the current and previous sections, it is evident that nutritional level and BW can influence the sexual maturation of chickens during the pullet to hen transition period. However, BW change (increase or decrease) might not be the only outcome of shifts in nutrient intake. Therefore, BW might be confounded by different carcass fat, lean, and mineral composition. For instance, body composition has changed over the past decades in broiler breeders. Intensive genetic selection for growth and lean tissue deposition (Zuidhof et al., 2014; Carney et al., 2022) and increase in the relative degree of feed restriction (Renema et al., 2007) have made modern broiler breeders leaner and more efficient, with less fat deposition compared to decades ago. Total body fat composition, for example, decreased around the time of photostimulation, from 15% in the early 1990s (Bennett and Leeson, 1990) to around 8% currently (Figure 2; Noetzold et al., 2024). Similarly, abdominal fat pad decreased from around 3% in the early 1990s (van Emous, 2015) to 1% or less in recent studies (Zuidhof, 2018; Afrouziyeh et al., 2021a). Interestingly, fat deposition in laying hens does not seem to have changed considerably over the years (Figure 3), with greater carcass composition differences due to feeding strategies and not so much due to their genetic advancement (Bahry et al., 2023; Noetzold et al., 2025). Therefore, there is a lack of summarized information on the relationship of adipose tissue deposition and its role in chicken reproduction. The following sections discuss adipose tissue effects on laying and broiler breeder hens.

#### 4.2.1 Fatness

The development of fat tissue in mammals has been well characterized. While lipid biosynthesis mainly happens in the adipose tissue of mammals, in chickens it occurs in the liver, followed by adipose tissue (Leveille, 1969). Lipids are then stored in different locations (visceral, subcutaneous, and intramuscular), and later utilized for lipoprotein synthesis, which occurs in the liver in response to E_2_ produced mainly by ovarian SWF (Williams and Sharp, 1977; van der Klein et al., 2020). Estradiol targets its intracellular receptors (ER-α and ER-β) inside the hepatocytes (Hanlon et al., 2022) and stimulates production of VLDLy and VTG. Therefore, chicken liver is an important organ involved in lipoprotein synthesis and ovarian follicular growth. Lipolysis in adipose tissue of chickens is done by a lipase specifically found in subcutaneous and abdominal fat, producing non-esterified fatty acids that are transported via blood to different body cells (Lee et al., 2009; Everaert et al., 2022).

In chickens, fat tissue deposition occurs predominantly at the visceral (abdominal fat) level but also in the skeleton and subcutaneous regions, the latter two representing around 30%–40% of the total carcass fat (Everaert et al., 2022). Interestingly, broiler breeders and laying hens have around 25 g of abdominal fat around photostimulation age (22 and 18 weeks, respectively), which represents 1% and 2% of their live BW, respectively (Hadinia et al., 2019; van Eck et al., 2024). This corresponds to only 15%–20% of the total carcass fat content in chickens. It is uncertain whether the adipose tissue from all the different locations (visceral, subcutaneous, and intramuscular) play an equal role in supporting reproduction or other body functions. Increased mRNA level and protein expression towards reproductive functions have been reported in adipocytes from the abdominal fat tissue (Resnyk et al., 2017; Bornelöv et al., 2018), and more recently, increased synthesis of VLDLy was correlated with increased abdominal fat content (Ma et al., 2024). Thus, visceral fat may play a larger role in reproduction compared to subcutaneous and intramuscular lipids. However, this hypothesis must be further investigated.

Lipids are an important part of yolk precursors (lipoproteins), where follicle (yolk) contains substantial amount of lipids (approximately 60%; Speake et al., 1998). Thus, it could be hypothesized that carcass fat level might affect reproduction of poultry as a direct substrate to yolk formation. In broiler breeders, impaired VLDLy diameter was observed with overfed breeders in the past, which was also responsible for lower follicular development and impaired egg production (Walzem et al., 1994). Uptake of VLDLy by follicles was decreased when excessive fat deposition was presented in the early stages of egg production in breeder lines selected for high fat deposition (Ma et al., 2024). These reports suggested that excessive lipids induced ovarian dysfunction through lipotoxicity with a complex mechanism that induces inadequate uptake of VLDLy by follicles and follicular atresia (Walzem et al., 1994; Chen et al., 2006; Walzem and Chen, 2014; Ma et al., 2024). If on one hand exaggerated fat deposition impaired egg production, insufficient fat deposition also does not provide birds with enough reserves to commence egg production (Zuidhof, 2018; van der Klein et al., 2018a; 2018b) or to sustain egg laying persistency (Afrouziyeh et al., 2021b; Heijmans et al., 2021; Noetzold et al., unpublished). Additionally, follicular selection and maturation in breeders were impaired in lean lines in the middle and late stages of egg production (Ma et al., 2024). Literature observations indicated that excessive or minimal carcass fat deposition impairs reproduction in poultry, where no specific “optimal” carcass fat level has been recommended previously. These observations predicted that future biological challenges related to fat deposition in meat-type chickens will occur if the fat deposition trend continues to decrease (Figure 2; Eitan et al., 2014; van Emous, 2015; Zuidhof, 2018; van der Klein et al., 2018a; 2018b; Afrouziyeh et al., 2021b; Heijmans et al., 2021; Carney et al., 2022; Noetzold et al., 2024).

The lack of carcass fat content might directly affect substrate availability (lipoproteins) for follicular growth (Alvarenga et al., 2011). In addition to the role as a substrate in reproduction, fat tissue has been lately considered a multifunctional organ related to sexual maturation in chickens by secretion of hormones and growth factors known as adipokines or adipocytokines. These adipokines are reported to play a role in several metabolic pathways in reproduction. Since the discovery of leptin (**LEP**) in mammals (Zhang et al., 1994), several adipokines have been identified, including adiponectin, apelin, chemerin, resistin, vaspin and visfatin (Kurowska et al., 2021). Although chicken LEP was discovered recently (Seroussi et al., 2016) and reviewed elsewhere (Hanlon et al., 2020), most studies done previously have relied on mammalian LEP administration. This brings some limitations to reliable conclusions of past studies with LEP and its role in the physiological reproduction of chickens. Leptin might not have an endocrine role in chickens as reported in mammals and is yet not reported to influence reproduction in chickens (Bernardi et al., 2024a). However, other adipokines might have distinct functions in avian species when compared to mammals and their physiological reproductive role. Therefore, the following discussion summarized the main adipokines that are hypothesized to play an important role in chicken reproduction.

##### 4.2.1.1 Adiponectin

Adiponectin was discovered soon after leptin (Scherer et al., 1995), and its role in chicken reproduction is not yet fully understood. Adiponectin is a signaling molecule produced in adipocytes and secreted into the blood (Maddineni et al., 2005). Also, its expression and receptors (adipoQ, adipoR1, and adipoR2) are found in many tissues in the chicken, including adipose, skeletal muscle, liver, anterior pituitary, hypothalamus, and ovary (Maddineni et al., 2005; Chabrolle et al., 2007; Zhang et al., 2017; Li C. et al., 2021). Plasma adiponectin levels have been examined in immature broilers (Hendricks et al., 2009; Ramachandran et al., 2013; Chen et al., 2021), mature turkey hens (Diot et al., 2015), and broiler breeder males and females from immature to mature period (Grandhaye et al., 2019). In most of these studies, adiponectin concentration in plasma decreased as birds aged. Furthermore, mRNA levels in adipose tissue were inversely related to abdominal fat pad in chickens (Tahmoorespur et al., 2010). This negative relationship between plasma level of adiponectin and adipose level has also been observed in mammals and no clear explanation has been suggested (Arita et al., 1999). Among the accepted hypothesis, it is possible that the variation between cell size during fat deposition might impact on adipocyte function, where cell hypertrophy might decrease circulating adiponectin (Engin, 2017), confirmed by the lower mRNA expression with adipocyte hypertrophy. This might occur because of insulin resistance in adipocytes with greater triglyceride stores compared to smaller and insulin sensitive adipocytes (Havel, 2004; Fang and Judd, 2018). Additionally, receptor levels also need to be considered as receptors determine cell sensitivity to adiponectin. On the other hand, a recent study in broiler breeders observed that increased adiponectin level corresponded to an increase in testosterone levels in males, but a decrease plasma adiponectin level corresponded to increased E_2_ levels in females (Grandhaye et al., 2019). Still, more research is needed to evaluate the specific changes of adiponectin in plasma of chickens.

Adiponectin is hypothesized to participate in the reproduction cascade of chickens due to its expression (and receptors) in the HPG axis. In male chickens, expression level of adiponectin receptors was greater in testis of sexually mature breeder roosters compared to immature males (Ocon-Grove et al., 2008). This was consistent in male chickens, where the level of AdipoR1 and AdipoR2 mRNA quantities were 8.3- and 9-fold higher in adult males compared to prepubertal males (Ramachandran et al., 2013). This suggests that adiponectin stimulates steroidogenesis and spermatogenesis in male chickens. Similarly, adiponectin was mainly expressed in ovary and speculated to stimulate the ovarian steroidogenesis in female chickens (Chabrolle et al., 2007; Ramachandran et al., 2013; Li J. et al., 2021). Li et al. (2021b) observed higher expressions of adipoR1 in smaller follicles of laying hens at 30 weeks of age (SWF > SYF > LYF). The same authors administered an adiponectin agonist in SYF collected from laying hens at 30 weeks of age and observed an increased mRNA expression of adipoR1 and adipoR2, increased P_4_ and decreased E_2_ by the granulosa cells, respectively (Li J. et al., 2021). Additionally, granulosa cells from F1 follicles expressed greater levels of adipoR1 and adipoR2 compared to other smaller follicles (Hadley et al., 2020). These results suggest that adiponectin might stimulate P_4_ production and the further hormonal cascade in the HGP axis. Additionally, adiponectin might affect different cells within the follicles. In a different study with laying hens, only the theca cells of follicles larger than 6 mm had expressed mRNA adiponectin whereas granulosa cells had only expressions of adiponectin receptors (Hadley et al., 2020). Similar results were found in turkey, where theca cells had greater mRNA adiponectin expression compared to granulosa cells (Diot et al., 2015). Further, Chabrolle et al. (2007) showed adiponectin expression in the theca cells, while the AdipoR1 was greater in granulosa cells when compared to theca cells. These results might indicate a paracrine role of adiponectin rather than only endocrine in the ovarian steroidogenesis. Implications of a paracrine role suggest that adiponectin might be tissue-specific and not related exclusively to the adipose tissue.

**FIGURE 5:**
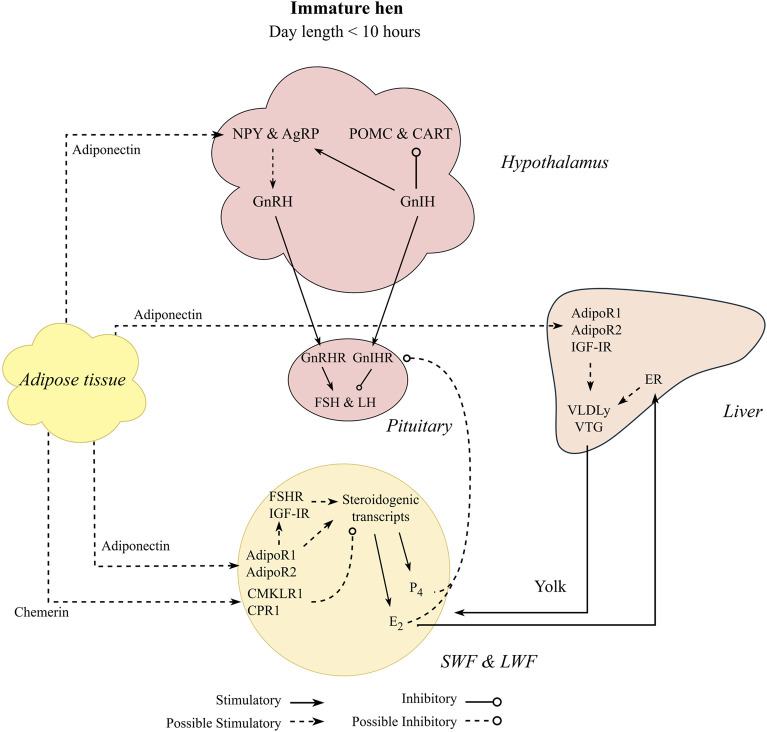
Proposed interaction between adipokines (adiponectin and chemerin) on the pullet to hen transition period. Pullets can initiate sexual maturation despite maintained under non-stimulatory photoperiod. In the small white follicles (SWF) and large white follicles (LWF), adiponectin is able to upregulate steroidogenesis by stimulating its receptors directly (AdipoR1 and AdipoR2) or through follicle stimulating hormone receptor (FSHR) and insulin-like growth factor I receptor (IGF-IR). Steroidogenic transcripts are involved in progesterone (P_4_) and estradiol (E_2_) production, which in turn negatively feedback on the gonadotropin inhibitory hormone receptor (GnIHR) at the pituitary. Adiponectin might also stimulate the orexigenic peptides (agouti-related peptide, AgRP, and Neuropeptide Y, NPY), which in turn stimulates gonadotropin inhibitory hormone (GnRH) release. Adiponectin effects on the liver might be related to lipoprotein production through its receptors or IGF-IR. On the other hand, chemerin is hypothesized to downregulate steroidogenic transcripts through activation of its receptors (CMKLR1 and CPR1) in the SWF and LWF. The dynamic effects of the adipokines after sexual maturation might change as the pre-hierarchical and pre-ovulatory follicles begin to emerge.

The stimulatory effects of adiponectin in ovarian development might occur through different pathways. First, adiponectin might stimulate follicular growth through steroidogenesis (Figure 5). Adiponectin agonist has increased STAR and Cyp19a1 expression in cultured granulosa cells (Li J. et al., 2021). Similar results were observed in mature geese (Meng et al., 2019). Additionally, a recent study showed that adiponectin agonist increased STAR, Cyp19a1, and Cyp11a1 expressions, along with increased FSHR in granulosa cells of SYF in laying hens (Li et al., 2025) and increased progesterone production. Interestingly, another study that administered an adiponectin agonist had steroidogenesis downregulated through STAR inhibition (Hadley et al., 2020). Most of these studies used cultured cells of mature laying hens, and differences might be attributed to the different molecules used as adiponectin agonists. Additionally, adiponectin might also be connected to appetite control (Figure 5). Fasting mature egg-type chickens for 48 h decreased adiponectin mRNA levels in the adipose tissue, liver, and pituitary gland (Maddineni et al., 2005). Also, adiponectin might downregulate fat deposition and promote energy expenditure (Maddineni et al., 2005; Yan et al., 2014). However, results showed that intracerebroventricular injection of adiponectin in layer chicks increased appetite, where the authors linked the hyperphagia to stimulation of the NPY receptor 1 (Madadi et al., 2023). Mellouk et al. (2018c) found differences in plasma adiponectin levels between restricted broiler breeders and breeders fed 1.7 times the restricted treatment from 3 to 9 weeks of age, whereas no differences were observed from 10 to 39 weeks of age. Furthermore, expression of AdipoR1 was higher in liver and intestinal tract of feed restricted when compared to unrestricted broilers (Cai et al., 2021). The authors indicated a possible role of adiponectin in metabolism of chickens, which is still not clear whether it is inhibitory (catabolism) or stimulatory (anabolism). We hypothesized that the plasma adiponectin circulating levels might not be the only factor that determines its physiological action (Figure 5). Although most studies indicate a decrease in plasma levels of adiponectin as birds increase fat deposition, its plasma level is still relatively high (Diot et all., 2015; Cai et al., 2021). More studies are needed to explain adiponectin expression and its receptors on the HPG axis of chickens, especially at the pullet to hen transition period. This might be a key factor in determining its physiological functions.

##### 4.2.1.2 Chemerin

In mammals, chemerin and its receptors have been expressed in several reproductive organs of females (Goralski et al., 2007; Singh et al., 2018). Its role is not fully understood, but it appears to regulate follicular development in mammals. Far less data is available for chemerin in chickens compared to adiponectin, and most of its apparent physiological role in chickens is based on studies with mammals. In turkeys, chemerin mRNA is more abundant in the liver than in heart and skeletal muscles, and also well expressed in ovarian cells (Diot et al., 2015). In chickens, chemerin expression is reported in ovary, adipose, muscle, and liver (Mellouk et al., 2018a). Chemerin receptors, Chemokine like receptor 1 (**CMKLR1**), and G protein-coupled receptor 1 (**GPR1**) have also been found in adipose, ovary, muscle, and liver (Diot et al., 2015; Mellouk et al., 2018b). Furthermore, chemerin was found in the oviduct portions of chickens and wild birds, with accumulation in the egg albumen (Estienne et al., 2022a; Estienne et al., 2022b; Bernardi et al., 2024a; Bernardi et al., 2024c).

In male chickens, human recombinant chemerin downregulated testosterone production of *in vitro* testis (Estienne et al., 2020). The mechanism was related to the steroidogenesis pathway, through lower STAR and 3-beta-hydroxysteroid dehydrogenase expressions (Estienne et al., 2020). In female chickens, although chemerin is hypothesized as a potential modulator of folliculogenesis (Bernardi et al., 2024a), its effects on chicken ovarian development are still unclear. Mellouk et al. (2018b) found that restricted feeding from 5 to 39 weeks in broiler breeders increased plasma chemerin levels during the initial laying period (21 weeks of age) and delayed sexual maturity compared to increased feeding treatment (1.7 times greater than restricted). These effects might be part of a cascading sequence and indicate a negative correlation between chemerin and sexual maturation age (Figure 5). The authors also correlated chemerin positively with increased F1 weight (Mellouk et al., 2018b). Despite that, feed intake level and fatness were not considered and might have also delayed sexual maturation rather than only chemerin (Mellouk et al., 2018c). Additionally, administration of chicken recombinant chemerin decreased progesterone production by *in vitro* pre-ovulatory follicles of broiler breeders (Bernardi et al., 2024b). Overall, a negative effect on reproduction is initially hypothesized by chemerin plasma levels and its expression of the *in vitro* conditions (Figure 5). Still, more information is needed regarding chemerin and its receptors on reproduction of female chickens with *in vivo* conditions.

## 5 Conclusion

In summary, it is evident that photostimulation is not the only factor that activates reproduction in commercial egg- and meat-type chickens. An integrative function between body composition and nutritional status of pullets is proposed, where the control mechanisms in the pullet to hen transition are likely highly conserved in egg- and meat-type chickens. While more information regarding the specific physiological pathways is needed, an opportunity for optimal nutritional and carcass fat level arises and can be useful to optimize reproductive performance of commercial chickens. Further elucidation of the control mechanisms will require additional evaluation of the HPG axis and tissues that are related to HPG axis during the transition period.
